# Reduced responses to glutamate receptor agonists follow loss of astrocytes and astroglial glutamate markers in the nucleus tractus solitarii

**DOI:** 10.14814/phy2.13158

**Published:** 2017-03-07

**Authors:** William T. Talman, Deidre Nitschke Dragon, Li‐Hsien Lin

**Affiliations:** ^1^ Laboratory of NeurobiologyDepartment of NeurologyCarver College of MedicineIowa CityIowa; ^2^ Department of Veterans Affairs Health Care SystemIowa CityIowa

**Keywords:** Astrocyte, baroreflex, glutamate, saporin

## Abstract

Saporin (SAP) or SAP conjugates injected into the nucleus tractus solitarii (NTS) of rats kill astrocytes. When injected in its unconjugated form, SAP produces no demonstrable loss of or damage to local neurons. However bilateral injections of SAP significantly attenuate responses to activation of baroreceptor reflexes that are mediated by transmission of signals through glutamate receptors in the NTS. We tested the hypothesis that SAP would reduce cardiovascular responses to activation of NTS glutamate receptors despite its recognized ability to spare local neurons while killing local astrocytes. In animals treated with SAP and SAP conjugates or, as a control, with the toxin 6‐hydroxydopamine (6‐OHDA), we sought to determine if dose‐related changes of arterial pressure (AP) or heart rate (HR) in response to injection into NTS of N‐methyl‐d‐aspartate (NMDA) or *α*‐amino‐3‐hydroxy‐5‐methyl‐4‐isoxazolepropionic acid (AMPA) were attenuated. Also we quantified changes in immunoreactivity (IR) for EAAT2, EAAC1, and VGluT2 in NTS after SAP and SAP conjugates. Our earlier studies showed that IR for NMDA and AMPA receptors was not changed after injection of SAP. We found that EAAT2 and EAAC1, both found in astrocytes, were reduced by SAP or its conjugates but not by 6‐OHDA. In contrast, VGluT2‐IR was increased by SAP or conjugates but not by 6‐OHDA. AP and HR responses to NMDA and AMPA were attenuated after SAP and SAP conjugate injection but not after 6‐OHDA. Results of this study are consistent with others that have shown interactions between astroglia and neurons in synaptic transmission mediated by glutamate receptor activation in the NTS.

## Introduction

Previously, we reported toxic cellular effects produced by injection of stabilized substance *P* conjugated with saporin (SSP‐SAP) or of an antibody to dopamine‐*β*‐hydroxylase (DBH) conjugated with saporin (anti‐DBH‐SAP) into the nucleus tractus solitarii (NTS). SSP‐SAP targeted and killed neurons that express the neurokinin 1 (NK1) receptor, while anti‐DBH‐SAP targeted and killed neurons that express DBH (Madden et al. 2006; Nayate et al. 2009; Talman and Lin 2013). Both toxins also killed NTS astrocytes and were associated with loss of cardiovascular responses to baroreflex activation (Lin et al. 2013). SAP alone injected into the NTS similarly led to death of astrocytes with no demonstrable damage to NTS neurons labeled with PGP9.5 (Lin et al. 2013). In contrast, injection of 6‐hydroxydopamine (6‐OHDA), a toxin that targets catecholamine neurons (Thoenen and Tranzer 1968), spared astrocytes (Lin et al. 2013). SAP, despite its sparing local neurons, led to loss of baroreceptor and other cardiovascular reflexes (Lin et al. 2013) whose signals are transmitted through NTS (Andresen and Yang 1990; Ciriello 1983; Kalia and Mesulam 1980; Palkovits and Zaborszky 1977). 6‐OHDA had no such cardiovascular effects (Lin et al. 2013). These findings suggested that damage confined to astrocytes altered transmission of reflex signals through the NTS but left open to question whether physiological responses to local activation of NTS neurons would be altered by SAP.

We have shown that NTS neurons that express NK1 receptors also express receptors for the excitatory amino acid glutamate (Lin et al. 2008), a transmitter released by baroreceptor reflex afferent nerve terminals in the NTS (Lawrence 1995; Lawrence and Jarrott 1994; Talman et al. 1980). As was expected, injection of SSP‐SAP not only led to loss of neurons expressing NK1 receptors but also to loss of cells expressing N‐methyl‐d‐aspartate (NMDA) receptors, and *α*‐amino‐3‐hydroxy‐5‐methyl‐4‐isoxazolepropionic acid (AMPA) receptors, both of which colocalize with NK1 receptors in NTS neurons (Lin et al. 2008). Therefore, reduced glutamate receptor‐mediated responses would be anticipated after injection of SSP‐SAP. In contrast, NMDA and AMPA receptors were not affected by injection of the catecholaminergic neuronal toxins anti‐DBH‐SAP or 6‐hydroxydopamine (6‐OHDA) or of unconjugated SAP (Lin et al. 2013; Talman et al. 2012). While SAP and each of the SAP conjugates also killed astrocytes in the NTS, 6‐OHDA did not. Therefore, if changes in responses to glutamate receptor activation were dependent on damage to neurons that expressed those receptors, such changes would not be expected after treatment with either anti‐DBH‐SAP or 6‐OHDA. However, knowing that baroreflexes, which are mediated through glutamate transmission in NTS (Colombari et al. 1997; Talman et al. 1981b), are attenuated by SAP or SAP conjugates and that astrocytes themselves uptake and release glutamate (Fremeau et al. 2002; Kimelberg et al. 1990; Rothstein et al. 1994) and participate in neuroglial interaction and synaptic function in the brain (Derouiche and Frotscher 1991; Huda et al. 2013), we hypothesized that SAP interferes with transmission through glutamate receptors in the NTS. Furthermore, we hypothesized that SAP conjugates or SAP alone, in killing local astrocytes, would lead to changes in glutamate transporter, a glial marker, and further support involvement of glia in excitatory transmission in the NTS (Huda et al. 2013).

## Materials and Methods

All studies using experimental animals conformed to standards established by the Guide for the Care for the Care and Use of Laboratory Animals (National Research Council, 2011) and were approved by the institutional animal care and use committees of the Iowa City Department of Veterans Affairs Medical Center and of the University of Iowa Carver College of Medicine. Adult male Sprague‐Dawley rats (250–300 gm; Harlan) were anesthetized with Isoflurane (5% for induction; 2% maintenance) delivered in 100% oxygen through a nasal mask. Depth of anesthesia was confirmed by assessing any motor, arterial pressure, or heart rate responses to tail pinch. Throughout experiments, the animal's body temperature was maintained at 37°C with a rectal probe and a heating pad connected to a temperature controller (YSI model 73A, Yellow Springs, OH). The animal was placed in Kopf stereotaxic frame (David Kopf Instruments, Tujunga, CA) and the brain stem was visualized through a partial occipital craniotomy. Drugs were microinjected under stereotaxic control through glass micropipettes into the NTS (0.4 mm rostral to the *calamus scriptorius*, 0.5 mm lateral to the midline, and 0.5 mm below the dorsal surface of the brain stem). As previously described, the following toxins dissolved in phosphate‐buffered saline (PBS) were microinjected into the NTS: SAP (3 ng in 100 nL, *N* = 6); SSP‐SAP (3 ng in 100 nL, *N* = 6); anti‐DBH‐SAP (42 ng in 200 nL, *N* = 7); or 6‐OHDA (1 *μ*g in 400 nL, *N* = 6). Doses and volumes of injectate were the same as used in our prior studies of each agent (Lin et al. 2013; Nayate et al. 2009; Rothstein et al. 1994; Talman and Lin 2013). PBS alone was injected into the NTS (control side) in the same volume as the toxin injected on the opposite side. Microinjections were made in 25 nL increments with 30 sec between each increment until the total volume was reached (100–400 nL). Buprenorphine hydrochloride (0.01–0.05 mg/kg) was given subcutaneously for analgesia 30 minutes prior to termination of anesthesia. Wounds were sutured closed, lidocaine (2–4%) drops were applied over the sutures, and animals were given ampicillin (20–50 mg/kg) intramuscularly. Animals were allowed to recover from surgery and remained in their home cage for 1 week before being returned to the laboratory for instrumentation and glutamate agonist testing. Animals were then anesthetized again with Isoflurane (2%), which was maintained throughout agonist testing. A cannula was placed in a femoral artery and connected to a PowerLab/8SP acquisition system (ADInstruments Inc., Colorado Springs CO) for recording arterial blood pressure (AP), mean arterial blood pressure (MAP), and heart rate (HR). The original craniotomy site was reopened and the cerebellum retracted to expose the brain stem for injection of increasing doses of the GLU agonists AMPA and NMDA (0.1, 1, 5, 10 pmols/25 nL) through glass micropipettes into the NTS. We varied the order of agonists but always made the initial injection on the control side where PBS had been injected previously. We allowed 5 minutes between injection of the complete volume of each dose and agent and the beginning of injection of the next dose or agent. The time allowed cardiovascular variables to return to basal levels before succeeding doses and agonists. After all four doses (0.1, 1, 5, 10 pmols/25 nL) of agonist had been given on the control side, the same four doses of agonist were injected on the side that had been treated with toxin. The other agonist was then injected on the control side followed by injections on the side treated with toxin. Upon completion of dose responses for each agonist, animals were killed with intraperitoneal injections of Nembutal (50 mg/kg),

For immunohistochemical analysis of the effect of toxins on glial glutamate transporter (EAAT2; also called GLT‐1 or excitatory amino acid transporter), neuronal glutamate transporters EAAC1 and vesicular glutamate transport type 2 (VGluT2) in the NTS (Rothstein et al. 1994), we injected each toxin unilaterally into adult male Sprague‐Dawley rats (280–330 g, *N* = 5 or 6 for each toxin) and killed and perfused the rats 7 days later under pentobarbital (50 mg/kg) anesthesia. The brain was then removed, post‐fixed in cold 4% paraformaldehyde in phosphate‐buffered saline (PBS) for 2 h and then cryo‐protected for 2 days in 30% sucrose in PBS at 4°C. Frozen 20 *μ*m coronal sections were cut with a cryostat and mounted on Colorfrost Plus microscope slides (Fisher Scientific, PA). Transverse brain stem sections containing NTS were processed for immunofluorescent staining using methods similar to those described in our previous publications (Lin et al. 2008). Briefly, sections were incubated in goat anti‐EAAC1 antibody (purchased from Millipore, catalog number AB1520, 1:500 dilution), Guinea pig anti‐EAAT2 (purchased from Millipore, catalog number AB1783, 1:1000 dilution), or Guinea pig VGluT2 (a gift from Dr. R. Edwards, 1:4000 dilution) in 10% donkey normal serum for 20–24 h in a humid chamber at 25°C. Anti‐EAAC1 antibody produced similar staining patterns in CNS as those described using in situ hybridization with probes to rEAAC1 mRNA and preabsorption of the antiserum with the immunogen peptide completely abolished the immunostaining (information provided by Millipore). Specificity of anti‐EAAT2 has been confirmed by western blot and also by preabsorption of the antiserum with immunogen peptide (information provided by Millipore). Anti‐VGluT2 antibody was generated using a peptide that corresponds to 29 amino acids in the C‐terminal of rat VGluT2 and its specificity has been reported (Fujiyama et al. 2001; Kaneko et al. 2002). Sections were then incubated with fluorescent secondary antibody made in donkey against the species from which the primary antibody was made (Jackson ImmunoResearch Labs, 1:200 dilution for all secondary antibodies) in PBS for 20–24 h at 4°C. Stained sections were cover‐slipped with Prolong Gold Anti‐fade Reagents (Invitrogen‐Molecular Probes) after the final washes with PBS. We examined stained sections with a Zeiss LSM 710 confocal microscope and obtained pseudocolored digital images. Negative controls consisted of tissue processed in the absence of primary antibodies.

Physiological data were reported as mean ± standard error of the mean (SEM). Physiological responses to agonists in each of the experimental settings (control, SSP‐SAP, anti‐DBH‐SAP, 6‐OHDA, and SAP) were analyzed using Analysis of Variance (ANOVA) with Tukey post hoc comparisons both between responses to agonists on the toxin‐treated side versus the control side and between doses. Significance was accepted at *P* ≤ 0.05.

We quantified changes in immunoreactivity (IR) in the NTS using NIH ImageJ software, a public domain program available from the National Institutes of Health (http://rsb.info.nih.gov/ij/). IR was expressed as gray value in arbitrary units and included all IR observed in cells and processes in the NTS. IR units per section were adjusted for differences in NTS size among sections. We randomly selected 2–3 sections of the NTS within 200 *μ*m of the center of the injection site for analysis. Uninjected NTS from the same section was used as a control. Anatomical landmarks were used as boundaries for the NTS area selected for analysis. Students *T*‐test was used to determine if IR in the NTS was significantly altered by toxin injection. Significance was accepted at *P* ≤ 0.05.

## Results

### Immunofluorescent analysis of EAAT2‐IR after SAP, SSP‐SAP, anti‐DBH‐SAP, and 6‐OHDA

**Figure 1:**
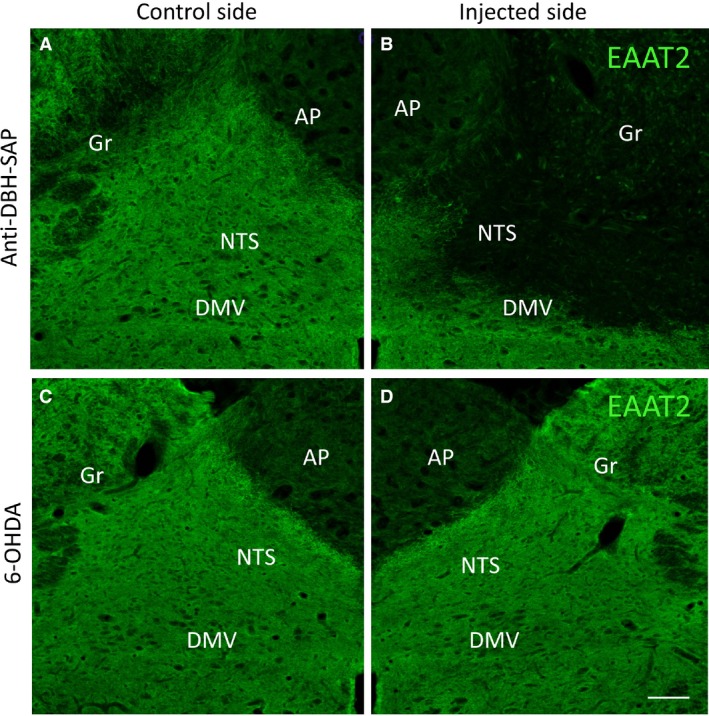
A decrease in EAAT2‐IR is noted in the NTS after anti‐DBH‐SAP injection on the right side (B) when compared with the opposite, control, side (A). In contrast, no change in EAAT2‐IR was noted after injection of 6‐OHDA into the NTS (D), when compared with the control side (C). Similar changes in EAAT2‐IR were also noted after injection of SAP or SSP‐SAP into the NTS (not shown). For this figure, as for Figures 2 and 3, an example of the histological changes produced by only one toxin is shown because images for SAP, SSP‐SAP, and anti‐DBH‐SAP are indistinguishable from each other. We chose to show a representative figure of the response to one toxin instead of showing repetitive identical images. AP, area postrema; DMV, dorsal motor nucleus of vagus; Gr, gracilis nucleus. Scale bar = 100 *μ*m.

There was a significant decrease in EAAT2‐IR in the NTS 7 days after injection of SAP (44.7 ± 7.2% of control; *P* < 0.001), of SSP‐SAP (36.6 ± 5.8% of control, *P* < 0.001, Fig. 1), and of anti‐DBH‐SAP (32.9 ± 5.8% of control, *P* < 0.001), while no change in EAAT2‐IR was noted after injection of 6‐OHDA. Figure 1B shows a representative decrease in EAAT2‐IR in the NTS 7 days after injection of anti‐DBH‐SAP when compared with the control side (Fig. 1A). In contrast, no change in EAAT2‐IR was noted after injection of 6‐OHDA (Fig. 1D) when compared to the control side (Fig. 1C).

### Immunofluorescent analysis of VGluT2 after SAP, SSP‐SAP, anti‐DBH‐SAP, and 6‐OHDA

**Figure 2:**
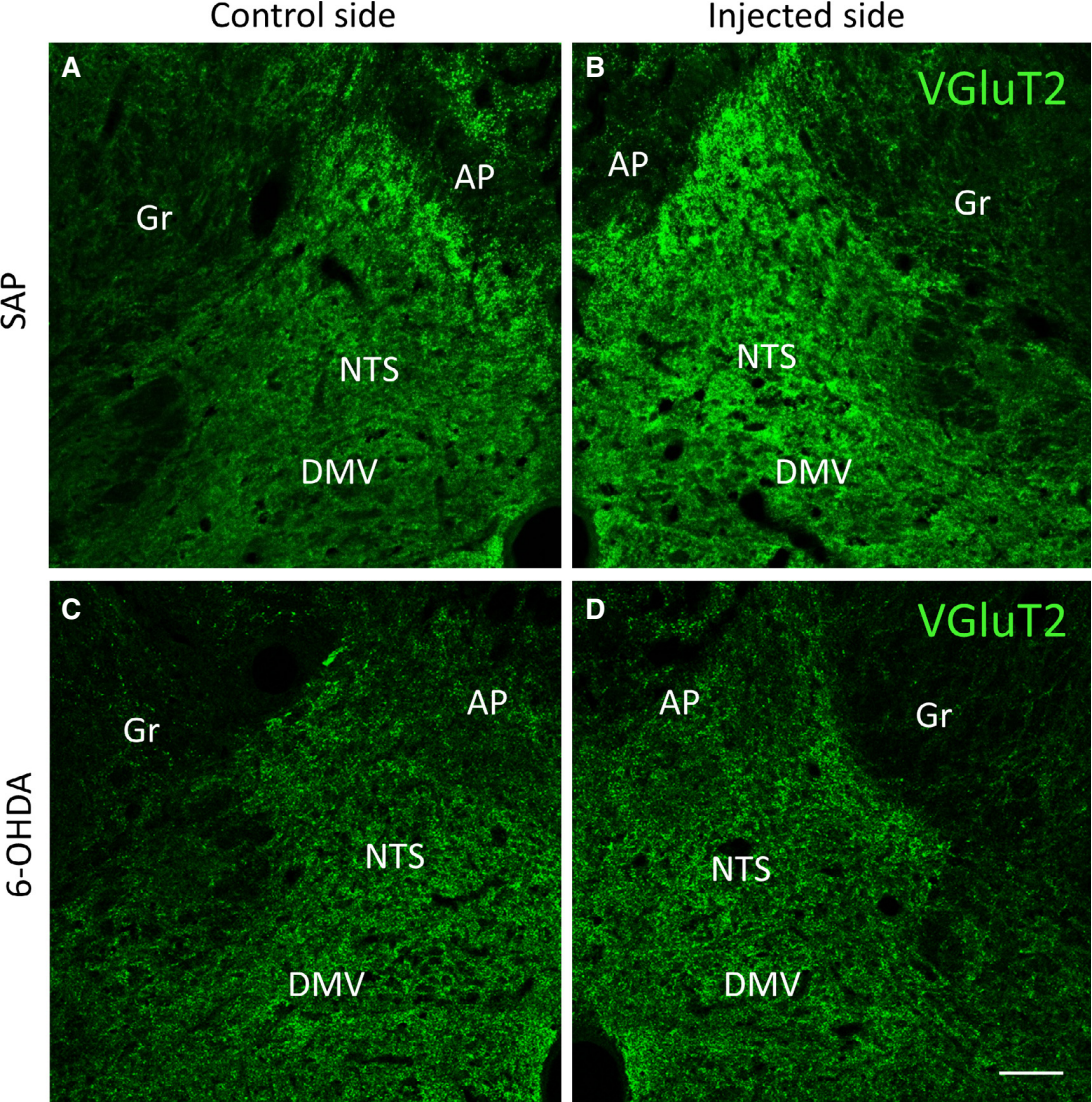
There is an increase in VGluT2‐ IR (B) in the NTS 7 days after SAP injection, when compared with VGluT2‐ IR on the control side (A). In contrast, no change in VGluT2‐IR was noted in the NTS after 6‐OHDA injection (D) when compared with that of the control side (C). Similar change in VGluT2‐IR was also observed after injection of SSP‐SAP or anti‐DBH‐SAP into the NTS (not shown). Abbreviations are as in Fig. 1. Scale bar = 100 *μ*m.

Seven days after injection of SAP, there was a significant increase (140.6 ± 16.2% of control, *P* < 0.001) in VGluT2‐IR in the NTS when compared with the control side. Increased VGluT2‐IR was also observed in the NTS following injection of SSP‐SAP (165 ± 28.5% of control, *P* < 0.001) and anti‐DBH‐SAP (150 ± 27.8% of control, *P* < 0.001), but there was no change in VGluT2‐IR in the NTS after 6‐OHDA injection. Figure 2B shows a representative increase in VGluT2‐IR in the NTS 7 days after injection of SAP when compared with the control side (Fig. 2A). In contrast, no change in VGluT2‐IR was noted after injection of 6‐OHDA (Fig. 2D) when compared with the control side (Fig. 2C).

### Immunofluorescent analysis of EAAC1‐IR after SAP, SSP‐SAP, anti‐DBH‐SAP, and 6‐OHDA

**Figure 3:**
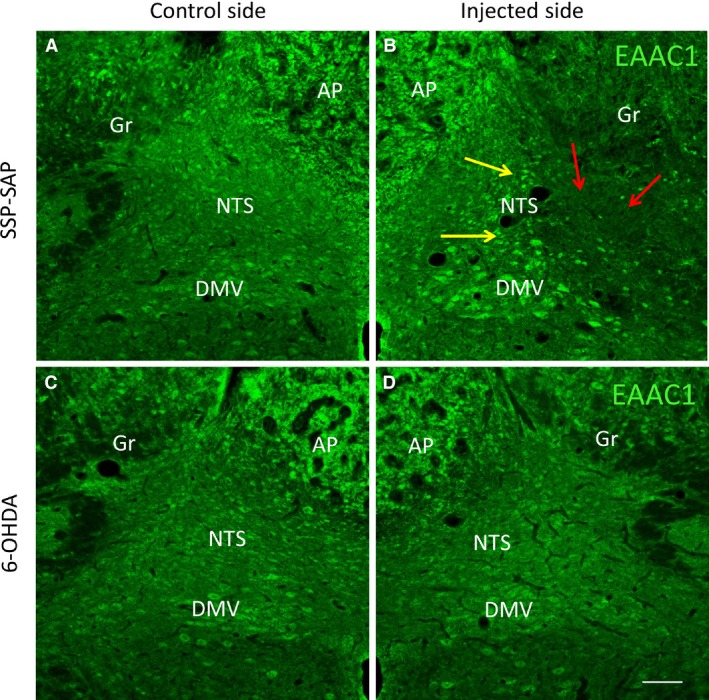
There is a decrease in EAAC1‐IR in the center of the injection (red arrows in B) and an increase in the peripheral area (yellow arrows in B) in the NTS after SSP‐SAP was injected into the NTS, as compared to that of the control side (A). No change in EAAC1‐IR was noted after 6‐OHDA injection (D), as compared to that of the control side (C). We observed similar changes in EAAC1‐IR after SAP or anti‐DBH‐SAP injection into the NTS (not shown). Abbreviations are as in Figure 1. Scale bar = 100 *μ*m.

When compared with the control side, 7 days after injection of SAP, SSP‐SAP, or anti‐DBH‐SAP into the NTS, there was decreased EAAC1‐IR at the center of the injection site but increased EAAC1‐IR on the periphery of the injection site. In contrast, no change in EAAC1‐IR was noted after 6‐OHDA injection. Statistical analysis of the composite changes was not performed because the decrease in the center and the increase in the periphery canceled each other out when quantified. However, analysis of findings as shown in the representative images (Fig. 3B) for effects of SSP‐SAP, showed that EAAC1‐IR at the center of the injection site was significantly decreased, while EAAC1‐IR on the periphery of the injection site was significantly increased when compared with the control side (Fig. 3A). In contrast, no change in EAAC1‐IR was noted after 6‐OHDA injection (Fig. 3D) when compared with the control side (Fig. 3C).

### Cardiovascular responses to NMDA after treatment with toxins

**Figure 4:**
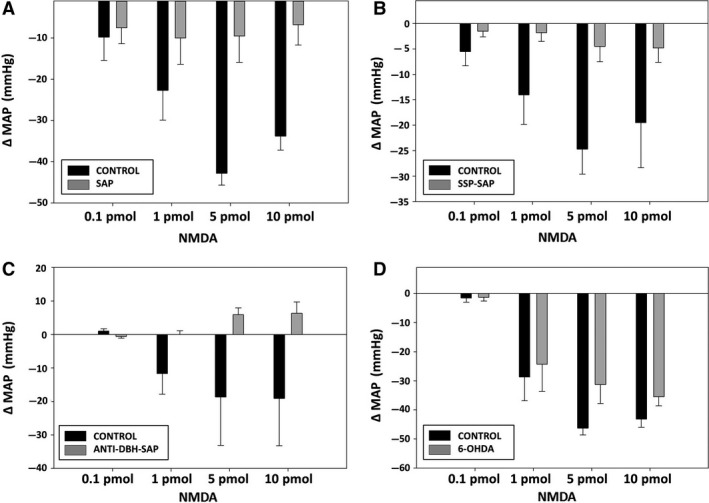
Reduced depressor responses to NTS injections of NMDA in animals after injection into the NTS of SAP (A), SSP‐SAP (B), and anti‐DBH‐SAP (C) but not after 6‐OHDA (D). Dose‐related responses of mean arterial pressure (MAP) to NMDA (0.1, 1, 5, 10 pmols/25 nL) were assessed 7 days after unilateral injection of each toxin with the untreated side of NTS serving as the control. SAP (*N* = 6; *P* < 0.0001); SSP‐SAP (*N* = 6; *P* < 0.0001); ant‐DBH‐SAP (*N* = 7; *P* < 0.001); 6‐OHDA (*N* = 6; NS). Basal MAP prior to NMDA injections, was 85 ± 4.0 mmHg in animals treated with SAP, 86 ± 2.8 mmHg in animals treated with SSP‐SAP, 93.0 ± 5.7 mmHg in animals treated with anti‐DBH SAP, and 89 ± 3.5 in animals treated with 6‐OHDA.

**Figure 5:**
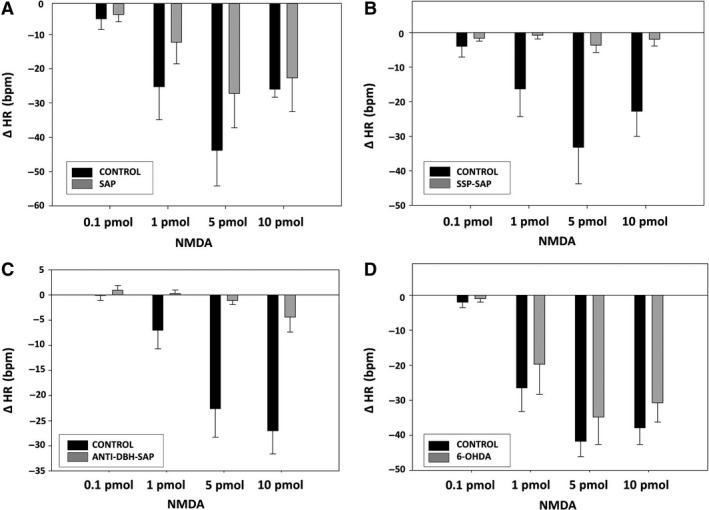
Reduced bradycardic responses to NTS injections of NMDA in animals after injection into the NTS of SAP (A), SSP‐SAP (B), and anti‐DBH‐SAP (C) but not after 6‐OHDA (D). Dose‐related responses of heart rate (HR) to NMDA (0.1, 1, 5, 10 pmols/25 nL) were assessed 7 days after unilateral injection of each toxin with the untreated side of NTS serving as the control. SAP (*N* = 6; NS); SSP‐SAP (*N* = 6; *P* < 0.0001); ant‐DBH‐SAP (*N* = 7; *P* < 0.0001); 6‐OHDA (*N* = 6; NS). Basal HR prior to NMDA injections was 332 ± 11.6 bpm in animals treated with SAP, 322 ± 11.2 bpm in animals treated with SSP‐SAP, 331 ± 8.5 bpm in animals treated with anti‐DBH SAP, and 333 ± 20.7 bpm in animals treated with 6‐OHDA.

Treatment of the NTS with SAP, SSP‐SAP, or anti‐DBH‐ SAP led to significantly reduced dose‐related MAP responses to NMDA microinjected into the NTS (Fig. 4). Dose‐related HR responses to NMDA were also significantly reduced after anti‐DBH‐SAP and SSP‐SAP (Fig. 5). However, HR tends to be more variable than AP; and, although SAP alone did decrease HR responses to NMDA, the change was not statistically significant. Treatment of the NTS with 6‐OHDA did not affect AP or HR responses elicited by NMDA (Figs. 4, 5).

### Cardiovascular responses to AMPA after treatment with toxins

**Figure 6:**
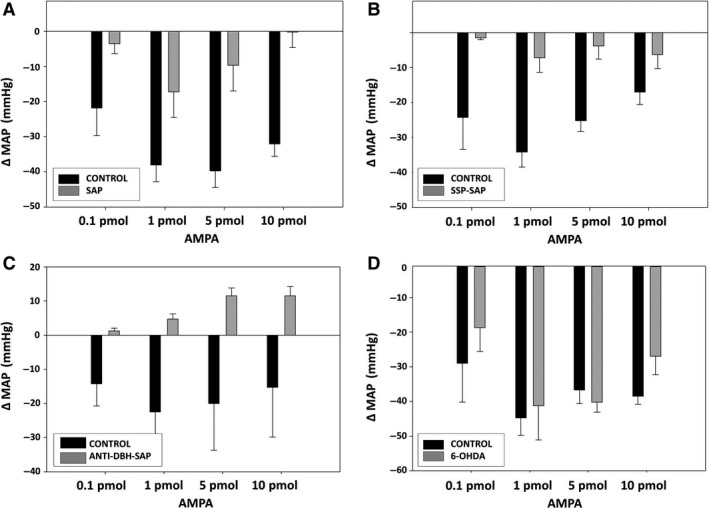
Reduced depressor responses to NTS injections of AMPA in animals after injection into the NTS of SAP (A), SSP‐SAP (B), and anti‐DBH‐SAP (C) but not after 6‐OHDA (D). Dose‐related responses of MAP to NMDA (0.1, 1, 5, 10 pmols/25 nL) were assessed 7 days after unilateral injection of each toxin with the untreated side of NTS serving as the control. SAP (*N* = 6; *P* < 0.0001); SSP‐SAP (*N* = 6; *P* < 0.0001); ant‐DBH‐SAP (*N* = 7; *P* < 0.0001); 6‐OHDA (*N* = 6; NS). Basal MAP prior to AMPA injections was 88 ± 3.2 mmHg in animals treated with SAP, 89 ± 2.5 mmHg in animals treated with SSP‐SAP, 93 ± 4.1 mmHg in animals treated with anti‐DBH SAP, and 85 ± 3.3 mmHg in animals treated with 6‐OHDA.

**Figure 7:**
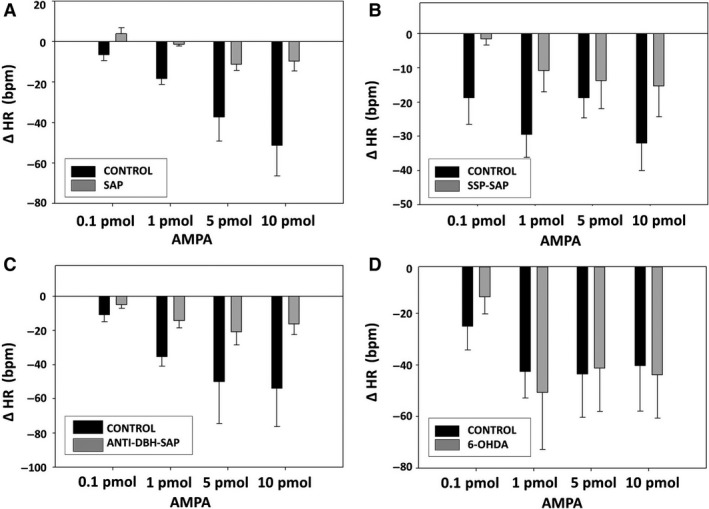
Reduced bradycardic responses to NTS injections of AMPA in animals after injection into the NTS of SAP (A), SSP‐SAP (B), and anti‐DBH‐SAP (C) but not after 6‐OHDA (D). Dose‐related responses of HR to NMDA (0.1, 1, 5, 10 pmols/25 nL) were assessed 7 days after unilateral injection of each toxin with the untreated side of NTS serving as the control. SAP (*N* = 6; *P* < 0.05); SSP‐SAP (*N* = 6; *P* < 0.01); ant‐DBH‐SAP (*N* = 7; *P* < 0.0001); 6‐OHDA (*N* = 6; NS). Basal HR prior to AMPA injections was 301 ± 11.8 bpm in animals treated with SAP, 326 ± 11.9 bpm in animals treated with SSP‐SAP, 331 ± 12.3 bpm in animals treated with anti‐DBH SAP, and 316 ± 14.2 bpm in animals treated with 6‐OHDA.

Treatment of the NTS with SAP or SAP conjugates also significantly reduced dose‐related AP responses (Fig. 6) and dose‐related bradycardic responses (Fig. 7) to AMPA microinjected into the NTS. 6‐OHDA again had no effect on AP or HR responses to AMPA (Figs. 6, 7).

## Discussion

This study demonstrates that SAP, a toxin that we have shown (Lin et al. 2013) selectively kills astrocytes with no demonstrable effect on neurons or neuronal glutamate receptors (GluR's) (Table 1), leads to decreased EAAT2 and EAAC1 at the center of the injection site but an increase in the vesicular glutamate transporter VGluT2 at the injection site. On the periphery of the injection site EAAC1‐IR was increased. In the context of demonstrated immunohistochemical changes, AP and HR responses both to AMPA and NMDA injected in the NTS were attenuated. Indeed, treatment of NTS with anti‐DBH‐SAP, which kills both astrocytes and catecholamine neurons, led to reversal of depressor/bradycardic responses with injection of glutamate receptor agonists so that, after that toxin, injection of the agonists led to pressor/tachycardic responses. The mechanism behind that reversal of responses is not known, but it would be fully consistent with the recognized difference in physiological involvement of minute regions of NTS so that stimulation of one such site could lead to pressor responses while stimulation of a proximate region could lead to depressor responses (Nelson et al. 1988). Therefore, a lesion that affected astrocytes and catecholamine neurons, which are confined to a small region of NTS (Dahlström and Fuxe 1964) and are involved in depressor/bradycardic responses to baroreceptor stimulation (Chalmers and Wurtman 1971), could lead to unopposed stimulation of pressor regions that lay nearby. Another potential explanation, which we find less tenable, is that agonists in the anti‐DBH‐SAP group crossed the midline to lead to bilateral depolarization blockade in the NTS with resulting pressor effects as seen with lesions of the NTS (Talman et al. 1981a). That explanation seems unlikely in that the pressor effects were themselves transient and reproducible, an unlikely occurrence with depolarization blockade (Lipski et al. 1988).

**Table 1:** Summary of IR changes in the NTS after injection of SAP, SAP conjugates, or 6‐OHDA

	SAP	SSP‐SAP	anti‐DBH‐SAP	6‐OHDA
NK1^a^	−	↓	−	−
NMDAR1^a^	−	↓	−	−
GluR2	−	↓^a^	−	−
GFAP^a^	↓	↓	↓	−
PGP9.5^a^	−	↓	−	−
DBH^a^	−	↑	↓	↓
TH^a^	−	↑	↓	↓
EAAT2	↓	↓	↓	–
VGluT2	↑	↑^a^	↑	–

Note that data for EAAC1‐IR were not included in this table because the IR in the center of the injection versus the periphery of the injection differed; therefore, even semi‐quantitation of changes in NTS regions that were homologous to those evaluated for the other peptides would have been misleading. ↓, Significant decrease (*P* < 0.05 or less); ↑, Significant increase (*P* < 0.05 or less); –, No significant change.

a Qualitative changes shown have been published previously (Lin et al. 2013, 2008, 2012; Talman et al. 2012).

The finding here that responses to glutamate receptor agonists injected into the NTS are attenuated after treatment with toxins that killed astrocytes in the same region is consistent with and complementary to earlier publications. Specifically, glutamate is considered a transmitter released by baroreceptor afferent nerves at their terminals in the NTS (Lawrence and Jarrott 1994; Talman et al. 1980) and toxins that kill astrocytes in the NTS lead to attenuation of the baroreflex (Lin et al. 2013), as would be expected with interruption of glutamate transmission in the NTS.

In that EAAT2 is found in astrocytes and colocalizes with glial fibrillary acidic protein (GFAP) (Milton et al. 1997), the loss of EAAT2 at the injection site is consistent with our earlier finding that GFAP is lost as a consequence of death of local astrocytes after SAP injection (Lin et al. 2013). Our previous studies have suggested that treatment with SAP causes selective loss of astrocytes. This study, on the other hand, could suggest that neurons were damaged in that IR for EAAC1, thought to be a neuronal transporter (Rothstein et al. 1994), was decreased at the center of the injection. However, it is well established that EAAC1 is found not only in neurons but also in GFAP‐positive astrocytes (Conti et al. 1998; Kugler and Schmitt 1999) and, therefore, would be expected to be reduced with loss of local astrocytes. Effects of the toxin on astrocytes are supported by enhanced EAAC1‐IR on the periphery of the injection site where, as we have previously shown, reactive astrocytes, expressing GFAP, concentrate (Lin et al. 2013). The enhanced EAAC1‐IR on the periphery may reflect its increase in neurons as has been found at the site of gliosis (De et al. 2016). As others have suggested (Chounlamountry et al. 2016), it is unlikely that changes in EAAC1 in the NTS participate in glutamate clearance or in excitation of glutamate receptors and are thus, an unlikely explanation for changes in physiological responses to activation of glutamate receptors as shown in this study.

We have previously shown (Table 1) that injection of SAP, anti‐DBH‐SAP, or SSP‐SAP, but not 6‐OHDA, leads to death of GFAP labeled astrocytes in the NTS (Lin et al. 2013); that anti‐DBH‐SAP or 6‐OHDA leads to loss of DBH neurons in the NTS (Talman et al. 2012); and that SSP‐SAP alone leads to loss of NK1 neurons in NTS (Nayate et al. 2009). Further, we have shown that SSP‐SAP, but not SAP, anti‐DBH‐SAP, or 6‐OHDA, leads to loss of NMDAR1‐IR and GluR2‐IR in the NTS (Lin et al. 2013) at the site of injection of the respective toxin. This finding might seem inconsistent with SAP's killing local GFAP‐positive astrocytes (Lin et al. 2013), which express both ionotropic as well as metabotropic glutamate receptors (Cai and Kimelberg 1997; Condorelli et al. 1993; Serrano et al. 2008). However, the greater intensity of staining for each of the glutamate receptors (GluR's) in neurons when compared with astrocytes would make it unlikely that loss of GluR‐IR in an area replete with neuronal GluR's would be identifiable.

This study also demonstrates (Table 1) that SAP leads to locally increased VGluT2, which is found both in neuronal somata and in terminals (Stornetta et al. 2003). This finding is in keeping with that of others (Antonucci et al. 2012) who spoke of there not being a loss of VGluT1 after treatment with saporin‐conjugated anti‐vesicular GABA transporter antibodies but showed (see their Fig. 8 B1) that VGlut1was increased in afferents in the region. Though that study only dealt with VGlut1, it seems relevant in that VGluT1, like VGluT2, is associated with glutamatergic neurons (Wojcik et al. 2004). Increased expression of VGLUT's at the lesion site would be consistent with sprouting of VGLUT‐containing afferent terminals at the site of interrupted tripartite synapses (Jones 1999) as a result of SAP‐induced loss of local astrocytes. It is doubtful that changes in VGlut2 in our study were responsible for the differences in responses to glutamate agonists in that neither glutamate release nor responsivity of neurons is affected when VGluT1 or VGluT2 has been knocked out (Ni and Parpura 2009).

This study provides evidence that is consistent with the previous suggestion that astroglia in NTS participate in excitatory transmission (Huda et al. 2013) and may do so through modulation of local responsivity to activation of specific glutamate receptors. In the absence of astroglia, responsiveness of neurons to activation of glutamate receptors could be due to a direct effect of glia on local tripartite synapses (Perea and Araque 2002) but could also be due to the effects on distant synapses (Fellin et al. 2006) and loss of the effects of astrocyte‐derived glutamate on extrasynaptic NMDA receptors (Fellin et al. 2004).

## Conclusions

The data from this study support the hypothesis that treatment with SAP would lead to reduced responses to glutamate receptor agonists in the NTS. The study supports an interdependence between intact astrocytes and neurons in physiological functions attributed to neurons and provide evidence for a mechanism that would explain why lesions confined to astrocytes lead to altered cardiovascular reflex control and, in some animals, asystolic sudden death (Lin et al. 2013).

## Conflict of Interest

None of the authors have a real or potentially perceived conflict of interest that could have in any way affected the outcome or reporting of this work.
